# Editorial: Advances in infectious disease research: tackling antimicrobial resistance, zoonoses, and more

**DOI:** 10.3389/fcimb.2025.1671915

**Published:** 2025-08-13

**Authors:** Priyanka Bhoj, Namdev Togre, Satish Rojekar, Lalit Batra, Nitin Amdare

**Affiliations:** ^1^ Lewis Katz School of Medicine, Temple University, Philadelphia, PA, United States; ^2^ Department of Pharmacological Sciences, Icahn School of Medicine at Mount Sinai, New York, NY, United States; ^3^ Center for Predictive Medicine for Biodefense and Emerging Infectious Diseases, University of Louisville, Louisville, KY, United States; ^4^ Department of Biochemistry, Albert Einstein College of Medicine, Bronx, NY, United States

**Keywords:** antimicrobial resistance, infectious diseases, multidrug resistant, clinical microbiology, microbiome

## Introduction

Infectious diseases are still a major global health challenge. The rise of antimicrobial resistance (AMR) and the frequent transfer of diseases from animals to humans are concerning trends. WHO has warned that AMR is *“one of the top global public health threats,”* contributing directly to the 1.27 million deaths in 2019 (Antimicrobial resistance). Likewise, studies indicate roughly 60% of emerging infectious diseases originate in animal hosts (Saba Villarroel et al., 2023). Together, these trends threaten to undermine decades of medical progress.

This Research Topic was conceived to bring together studies addressing these challenges. We sought diverse approaches—epidemiology, clinical, genomics, and molecular analysis—to deepen our understanding of pathogens, resistance mechanisms, and novel interventions. The five published articles span a range of pathogens, clinical settings, and analytical techniques. Below we synthesize their key findings thematically, highlighting advances in AMR surveillance, pathogen characterization, and broader insights.

## Antimicrobial resistance mechanisms and clinical surveillance

Habib et al. decoded the *mexB* efflux pump in *Pseudomonas aeruginosa* from implant-associated infections. They found high resistance rates against cefoperazone, gentamicin, and amikacin (64–67%). In total 57% and 12% of isolates were multidrug-resistant (MDR) and extensively drug-resistant (XDR), respectively. Most resistant strains carried *mexB* genes, implicating an active efflux system. Indeed, adding the efflux inhibitor, carbonyl cyanide m-chlorophenyl hydrazone, dramatically lowered minimal inhibitory concentrations of antibiotics, confirming pump-mediated resistance. The authors emphasize rigorous infection control and surveillance in hospital settings to prevent the spread of such efflux-driven MDR *P. aeruginosa*.

Ding et al. performed whole-genome sequencing (WGS) of 282 *Mycobacterium tuberculosis* isolates from Shenzhen, China, to map transmission and resistance. They reported MDR rates of 22.3% by phenotypic testing and 34.5% by molecular (WGS-based) testing, with retreatment patients showing high resistance. Sequencing uncovered 92 transmission clusters. Notably, 80% of these clusters shared identical drug-resistance mutations across all samples, indicating resistant strain transmission. This study demonstrates that WGS surveillance can reveal fine-scale tuberculosis (TB) transmission dynamics and underscores the urgency of tracking and treating MDR-TB.

Khan et al. retrospectively analyzed 279 wound infections in a Chinese hospital (2022–2024). They isolated 33 species by culture: 50.8% were Gram-positive (80.2% *Staphylococcus aureus*) and 41.2% Gram-negative (22.6% *P. aeruginosa*). Alarming, 82.4% of all isolates were resistant to at least one antibiotic. The study also identified infection-specific host biomarker patterns. The authors conclude that MDR bacteria are highly prevalent in wound infections in China and encourage for AMR awareness campaign, community hygiene, and education to curb their spread.

In the pediatric oncology setting, Murshed et al. examined 202 bloodstream infections (BSIs) in 145 immunocompromised children over four years. Gram-positive bacteria predominated (58.4% cases), chiefly coagulase-negative staphylococci, while Gram-negatives accounted for 41.0%. Most BSIs occurred during neutropenic induction chemotherapy. Antimicrobial susceptibility testing revealed concerning resistance: many Gram-positive isolates showed high resistance to penicillin and oxacillin, and Gram-negative organisms were frequently MDR to multiple antibiotic classes. The study underscores the need for site-specific surveillance and tailored antibiotic policies in high-risk wards.

## Microbial communities and pathogen ecology

Zhang et al. investigated the biliary microbiota associated with pigmented gallstones, using 16S rRNA sequencing of bile and gallstone samples. They identified 10 genres that were consistently abundant in both bile and stones. Notably, *Actinomyces*, *Streptococcus*, and *Achromobacter* were significantly more abundant in gallstones than in bile. Further analysis revealed 32 bacterial species carrying *β-glucuronidase* or *phospholipase* encoding genes (*uidA*, *pldA*, *plc*) for enzymes that can deconjugate bilirubin and contribute to pigment formation. *β-glucuronidase* producing *Streptococcus* spp. and *Parabacteroides merdae* (harboring both *uidA* and *pldA*) were key contributors to pigmented stone formation. This novel finding suggests that certain gut-derived bacteria and their enzymes may drive gallstone pathogenesis, opening avenues for new preventive or therapeutic strategies targeting the microbiome.

While not focused on AMR or zoonoses per se, this microbiome study exemplifies the “and more” breadth of our Research Topic. It highlights how examining microbial communities can uncover unexpected and evolving disease factors. Such studies may inform holistic approaches, such as targeting microbial enzymes, to prevent chronic infections.

## Future directions

Taken together, the insights from this topic highlight both advancements made and ongoing challenges that remain. First, the reported high resistance rates make clear the urgent need for new antimicrobials, diagnostics, and vaccines. WHO emphasizes that enhanced AMR surveillance and research into novel therapies are global priorities (Antimicrobial resistance). For instance, conserved protein regions in efflux pumps identified by Habib et al. could guide the development of pump inhibitors or rapid molecular tests, while WGS-based detection of mixed TB infections (Ding et al.) could guide personalized treatment to prevent resistance development. Continued investment in antimicrobial R&D, along with robust stewardship, remains crucial.

Second, the articles highlight specific gaps for future work. For example, several studies noted surprising levels of resistance in ostensibly “rare” pathogens or commensals, suggesting that even our clinical diagnostics may undercount the true burden. Enhanced culture methods, metagenomics, and point-of-care tools can improve detection. Moreover, understanding the factors that allowed resistant strains to spread in these cohorts (infection control lapses, antibiotic usage patterns) warrants further study. Promoting hygiene, sanitation, and prudent use of antibiotics along with investment in training, data sharing, and global surveillance infrastructure to monitor AMR in humans, animals, and the environment will help ensure we stay ahead of evolving microbes.

## Conclusion

The articles in this Research Topic make important advances in our understanding of infectious disease threats. Diverse pathogens and settings—from wound and blood infections to TB—show widespread resistance mediated by mechanisms, such as efflux pumps or target mutations. Moreover, they introduce innovative approaches (genomic, immunological, and ecological) to study them. We thank all contributing authors, reviewers, and topic co-editors—Drs. Priyanka Bhoj, Namdev Togre, Satish Rojekar, Lalit Batra, and Nitin Amdare—for their valuable work. As these findings are disseminated, we remain hopeful that the combined efforts of the research community will continue to drive progress against AMR, zoonoses, and other pressing infectious challenges.
